# Supplementary material for: Systems genetics identifies a role for Cacna2d1 regulation in elevated intraocular pressure and glaucoma susceptibility
Source: Nat Commun. 2017 Nov 24;8:1755. doi: 10.1038/s41467-017-00837-5 (PMC5701146; doi:10.1038/s41467-017-00837-5)
Supplement: Supplementary file 4 — Supplementary Information [file 41467_2017_837_MOESM4_ESM.pdf]

SI GUIDE

File Name: Supplementary Information
Description: Supplementary Figures and Supplementary Tables.

Supplementary Data

File Name: Supplementary Data 1
Description: Gene set enrichment analysis (GSEA) input

File Name: Supplementary Data 2
Description: All sequence polymorphisms and structural variants (insertions/deletions) between the reference sequence (B6) and parental strain D2 according to the Sanger Insititute's Mouse Genomes Project and the CITG.

File Name: Peer Review File

Supplementary Figure 1: Genetic interval mapping of IOP revealed a single significant eQTL on proximal Chr 5 (enclosed in black box) in BXD strains that are WT/WT and WT/MUT or MUT/WT at *Tyrp1* and *Gpnmb.* The presence of either or both mutations does not influence the location to which IOP maps.

A. Genetic interval mapping using revealed a single significant eQTL on proximal Chr 5 for IOP in BXD strains that are WT/WT at *Tyrp1* and *Gpnmb*.

B. Genetic interval mapping using revealed a single significant eQTL on proximal Chr 5 for IOP in BXD strains that are lacking both mutations in *Tyrp1* and *Gpnmb* (i.e., WT/MUT or MUT/WT).

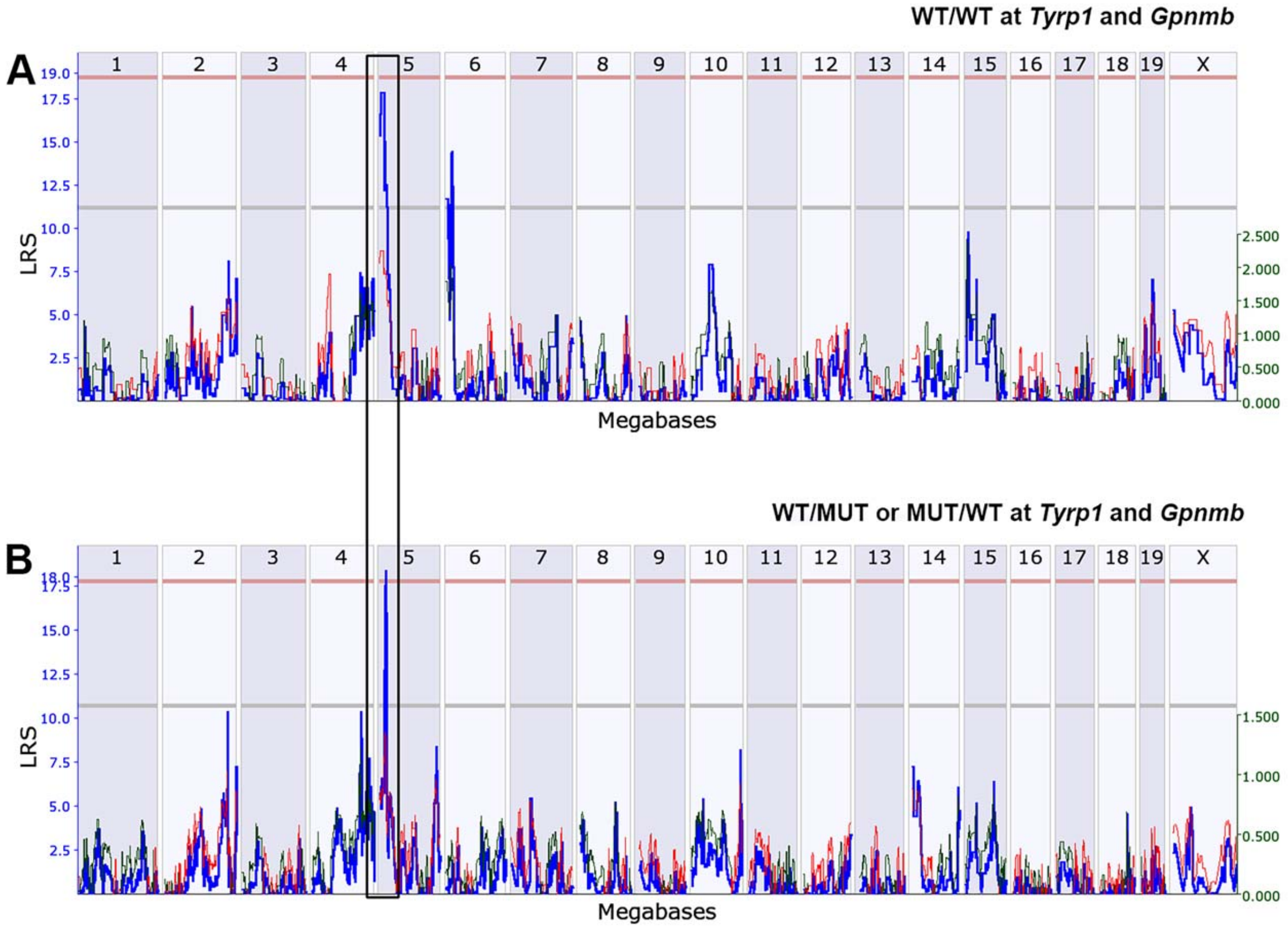

Supplementary Table 1: *Cis*-regulated positional candidates in the chromosome 5 locus arranged by location

Gene Symbol	Description	SNP count	Trait ID in GeneNetwork	Location	Max LRS
Sema3e	Sema domain, immunoglobulin domain (Ig), short basic domain, secreted, (semaphorin) 3E	67	1427673_a_at	Chr5: 14.2	15.0
Pclo	Presynaptic cytomatrix protein	503	1455373_at	Chr5: 14.8	28.2
Cacna2d1	Calcium channel voltage dependent alpha2/delta subunit 1	1056	1446324_at, 1449999_a_at	Chr5: 15.8	143.0
Gnai1	Guanine nucleotide binding protein, alpha inhibiting 1	14	1454959_s_at	Chr5: 17.7	19.4
Ptpn12	Protein tyrosine phosphatase, non-receptor type 12	19	1455105_at<br>1422045_a_at<br>1450479_x_at	Chr5: 20.5	14.8
Gsap	Gamma-secretase activating protein	6	1435596_at	Chr5: 20.8	23.0
Dnajc2	DnaJ (Hsp40) homolog subfamily C member 2	180	1417657_s_at	Chr5: 21.3	28.6
Psmc2	Proteasome 26S subunit ATPase 2	80	1426611_at<br>1435859_x_at<br>1443307_at	Chr5: 21.3	31.8
Lhfpl3	Lipoma HMGIC fusion partner-like 3	5633	1429592_at	Chr5: 22.6	16.1
Kmt2e	Lysine (K)-specific methyltransferase 2E	230	1432602_at	Chr5: 22.9	43.3
Srpk2	Serine/arginine-rich protein specific kinase 2	1024	1417134_at<br>1435746_at<br>1442241_at<br>1431372_at	Chr5: 23.0	142.6
Tomm7	Translocase of outer mitochondrial membrane 7 homolog	21	1428216_s_at	Chr5:23.3	106.1
Drctnnb1a	Down-regulated by catenin beta 1 activated	748	1450724_at	Chr5: 23.4	15.9
Klhl7	Kelch-like 7	343	1428091_at<br>1452660_s_at	Chr5: 23.6	31.4
Nupl2	Nucleoporin like 2	123	1456773_at	Chr5: 23.6	28.2
Nos3	Nitric oxide synthase 3	149	1422622_at	Chr5: 23.8	162.2
Abcb8	ATP-binding cassette, sub-family B (MDR/TAP), member 8	133	1423713_at	Chr5: 23.9	31.9

Accn3	Amiloride-sensitive cation channel 3	13	1439983_a_at	Chr5: 23.9	15.7
Slc4a2	Solute carrier family 4 (anion exchanger), member 2	77	1416637_at	Chr5: 23.9	31.3
Fastk	Fas-activated serine/threonine kinase	11	1460635_at	Chr5: 23.9	13.5
Smarcd3	SWI/SNF related, matrix associated, actin dependent regulator of chromatin, subfamily d, member 3	29	1418467_at	Chr5: 24.0	34.9
Crygn	Crystallin, gamma N	67	1425286_at	Chr5: 24.3	15.8
Abcf2	ATP-binding cassette, sub-family F (GCN20), member 2	52	1423863_at	Chr5: 24.6	14.4
Nub1	Nedd8 ultimate buster 1	180	1452777_a_at<br>1452776_a_at<br>1420535_a_at<br>1428439_at	Chr5: 24.7	110.0
Galnt11	UDP-N-acetyl-alpha-D galactosamine:polypeptide N-acetylgalactosaminyltransferase 11	9	1424748_at	Chr5: 24.7	55.5

Note: Whole eye transcript data from BXD strains were used to perform eQTL mapping for genes under the Chr 5 QTL locus. Those genes that had a peak LRS at the location of the gene (*cis* or locally regulated genes) were considered to be *cis*-regulated. Only the probes that hybridized to SNP-free regions of the genome were evaluated.

Key: LRS, likelihood ratio statistic

Supplementary Table 2: Positional candidates in the chromosome 5 locus and scoring

Gene Symbol	Criterion 1	Criterion 2	Criterion 3		Criterion 4	Criterion 5	Criterion 6	Criterion 7	Criterion 8	Score
	Located in Chr 5 QTL peak	*Cis*-eQTL	Linear correlation with IOP	Partial correlation with IOP	Functional network	Sequence variants in gene	Expression in eye structures associated with IOP	Human GWAS	Biological association with glaucoma or treatment	
Cacna2d1	YES	YES	YES	YES	YES	YES	YES	YES	YES	8
Ptpn12	YES	YES	YES	NO	-	-	-	-	-	3
Sema3e	YES	YES	YES	NO	-	-	-	-	-	3
Abcb8	YES	YES	NO	-	-	-	-	-	-	2
Abcf2	YES	YES	NO	-	-	-	-	-	-	2
Accn3	YES	YES	NO	-	-	-	-	-	-	2
Crygn	YES	YES	NO	-	-	-	-	-	-	2
Dnajc2	YES	YES	NO	-	-	-	-	-	-	2
Drctnnb1a	YES	YES	NO	-	-	-	-	-	-	2
Fastk	YES	YES	NO	-	-	-	-	-	-	2
Galnt11	YES	YES	NO	-	-	-	-	-	-	2
Gnai1	YES	YES	NO		-	-	-	-	-	2
Gsap	YES	YES	NO	-	-	-	-	-	-	2
Klhl7	YES	YES	NO	-	-	-	-	-	-	2
Kmt2e	YES	YES	NO	-	-	-	-	-	-	2
Lhfpl3	YES	YES	NO	-	-	-	-	-	-	2
Nos3	YES	YES	NO	-	-	-	-	-	-	2
Nub1	YES	YES	NO	-	-	-	-	-	-	2
Nupl2	YES	YES	NO	-	-	-	-	-	-	2
Pclo	YES	YES	NO	-	-	-	-	-	-	2

Psmc2	YES	YES	NO	-	-	-	-	-	-	2
Slc4a2	YES	YES	NO	-	-	-	-	-	-	2
Smarcd3	YES	YES	NO	-	-	-	-	-	-	2
Srpk2	YES	YES	NO	-	-	-	-	-	-	2
Tomm7	YES	YES	NO	-	-	-	-	-	-	2
Armc10	YES	NO	-	-	-	-	-	-	-	1
Asb10	YES	NO	-	-	-	-	-	-	-	1
Atg9b	YES	NO	-	-	-	-	-	-	-	1
CD36	YES	NO	-	-	-	-	-	-	-	1
Cdk5	YES	NO	-	-	-	-	-	-	-	1
Centg3	YES	NO	-	-	-	-	-	-	-	1
Fbxl13	YES	NO	-	-	-	-	-	-	-	1
Fgl2	YES	NO	-	-	-	-	-	-	-	1
Gbx1	YES	NO	-	-	-	-	-	-	-	1
Gnat3	YES	NO	-	-	-	-	-	-	-	1
Hgf	YES	NO	-	-	-	-	-	-	-	1
Kcnh2	YES	NO	-	-	-	-	-	-	-	1
Lrrc17	YES	NO	-	-	-	-	-	-	-	1
Magi2	YES	NO	-	-	-	-	-	-	-	1
Mll5	YES	NO	-	-	-	-	-	-	-	1
Napepld	YES	NO	-	-	-	-	-	-	-	1
Orc5l	YES	NO	-	-	-	-	-	-	-	1
Pmpcb	YES	NO	-	-	-	-	-	-	-	1
Pus7	YES	NO	-	-	-	-	-	-	-	1
Reln	YES	NO	-	-	-	-	-	-	-	1

Rheb	YES	NO	-	-	-	-	-	-	-	1
Rint1	YES	NO	-	-	-	-	-	-	-	1
Rsbn1l	YES	NO	-	-	-	-	-	-	-	1
Sema3a	YES	NO	-	-	-	-	-	-	-	1
Sema3c	YES	NO	-	-	-	-	-	-	-	1
Sema3d	YES	NO	-	-	-	-	-	-	-	1
Slc26a5	YES	NO	-	-	-	-	-	-	-	1
Speer3	YES	NO	-	-	-	-	-	-	-	1
Speer4c	YES	NO	-	-	-	-	-	-	-	1
Speer4d	YES	NO	-	-	-	-	-	-	-	1
Speer4e	YES	NO	-	-	-	-	-	-	-	1
Speer4f	YES	NO	-	-	-	-	-	-	-	1
Speer7-ps1	YES	NO	-	-	-	-	-	-	-	1
Speer8-ps1	YES	NO	-	-	-	-	-	-	-	1
Tmem60	YES	NO	-	-	-	-	-	-	-	1
Tmub1	YES	NO	-	-	-	-	-	-	-	1
Wd486	YES	NO	-	-	-	-	-	-	-	1

Note: Genes located under the Chr 5 QTL peak were scored sequentially based upon our 8 step selection criteria. If a gene did not fulfill a criterion (indicated by a dash), it was eliminated as a gene candidate for modulating IOP.

Supplementary Table 3: Effect of SNPs in *Cacna2d1* gene locus in NEIGHBORHOOD POAG case/control GWAS and multi-ethnic meta-analysis IOP GWAS

**NEIGHBORHOOD POAG case/control GWAS (Bailey et al., Nature Genetics, 2016 Feb;48(2):189-94.**											
CHR	BP	SNP	Allele 1	Allele 2	Freq1	Effect (beta)	StdErr	OR	p-value	n-studies	Direction
7	82011270	7:82011270:AATAC	d	r	0.2127	-0.1426	0.0432	0.86710084	0.0009616	8	--------
7	82011353	rs2299184	a	g	0.7798	0.1359	0.0413	1.145567331	0.001009	8	++++++++
7	82011262	7:82011262:TATAA	d	r	0.2226	-0.1328	0.042	0.875640203	0.001581	8	--------
7	82011505	rs2299185	a	g	0.2284	-0.1296	0.0412	0.878446739	0.001651	8	--------
7	82011839	rs6955149	a	g	0.2264	-0.1273	0.0413	0.880469492	0.002054	8	--------
7	82013761	rs11971283	c	g	0.1468	-0.1344	0.049	0.874240299	0.006075	8	----+---
7	82011004	7:82011004:G_GA	i	r	0.1462	-0.132	0.0488	0.876340995	0.006847	6	+---+---
7	82013521	rs11982475	t	c	0.8531	0.1301	0.0486	1.138942272	0.007447	8	-+++-+++
7	82010067	rs62465968	c	g	0.1395	-0.1293	0.0495	0.878710313	0.009054	6	+---+---
7	82013917	7:82013917:TGAA_	d	r	0.1425	-0.1253	0.0503	0.882232193	0.01285	8	----+---
7	81976258	rs117054034	t	c	0.9456	0.2484	0.1032	1.281972619	0.01606	3	?+++????
7	81971354	rs149421676	t	c	0.0543	-0.2476	0.1033	0.78067215	0.01653	3	?---????
7	82013962	7:82013962:G_GA	i	r	0.1515	-0.1202	0.0504	0.88674307	0.01699	8	+---+---
7	81799213	rs38541	a	c	0.722	0.0905	0.0398	1.094721508	0.02298	7	?+-+++++
7	81668864	rs73151509	t	c	0.0623	0.1694	0.0767	1.184593882	0.02712	7	?+++++++
7	81799650	rs38542	t	g	0.7164	0.0841	0.0392	1.087737662	0.03175	7	?+-+++++
7	82013724	rs10274659	t	c	0.7589	0.086	0.0403	1.089806328	0.03297	8	-+-+++++
7	81713810	rs73151543	t	c	0.9045	-0.1835	0.0862	0.832351876	0.03337	3	?--+????
7	81849719	rs146555498	t	c	0.9482	-0.2183	0.1063	0.80388424	0.04008	2	?-+?????
7	81806942	rs3919483	t	g	0.7259	0.0817	0.0399	1.085130222	0.04049	7	?+-+++++
7	81737230	rs59891073	c	g	0.086	-0.1259	0.062	0.881703013	0.04219	8	----+-+-
7	82009164	rs10954678	c	g	0.5887	0.0696	0.0343	1.072079264	0.04267	6	++++++--
7	82009771	rs10808299	a	c	0.4139	-0.0689	0.0341	0.933420017	0.04328	6	------++
7	81739755	rs78203609	t	c	0.9141	0.124	0.0614	1.132015871	0.04338	8	++++-+-+
7	81736414	rs6950805	a	t	0.9133	0.125	0.0621	1.133148453	0.04409	8	++++-+-+
7	81741120	rs77007284	a	g	0.9141	0.1229	0.0613	1.130771338	0.04482	8	++++-+-+
7	81736679	rs78489742	a	c	0.0868	-0.1242	0.062	0.883203183	0.04515	8	----+-+-
7	81736674	rs77787735	a	g	0.9132	0.1242	0.062	1.132242297	0.04518	8	++++-+-+
7	81736521	rs74521466	c	g	0.0868	-0.1242	0.062	0.883203183	0.04523	8	----+-+-
7	81736895	7:81736895:AAAT_	d	r	0.0868	-0.124	0.0619	0.883379841	0.04526	8	----+-+-
7	81736865	rs77450603	a	t	0.9132	0.124	0.0619	1.132015871	0.04531	8	++++-+-+
7	81737600	rs60993894	a	g	0.0867	-0.1236	0.0617	0.883733263	0.04532	8	----+-+-
7	81736729	rs74484242	a	c	0.0868	-0.124	0.062	0.883379841	0.04535	8	----+-+-
7	81738857	rs2097913	t	c	0.9137	0.1225	0.0612	1.13031912	0.04535	8	++++-+-+
7	81738190	rs79083404	a	g	0.9133	0.1231	0.0615	1.130997515	0.04537	8	++++-+-+
7	81738147	rs75530395	c	g	0.0867	-0.1231	0.0615	0.884175241	0.04546	8	----+-+-
7	81738919	7:81738919:TA_T	d	r	0.0863	-0.1224	0.0612	0.88479438	0.04546	8	----+-+-
7	81738984	rs75681972	a	g	0.0868	-0.1223	0.0612	0.884882864	0.04565	8	----+-+-
7	81737040	rs147041350	t	c	0.0868	-0.1237	0.0619	0.883644895	0.04573	8	----+-+-
7	81739027	rs78816329	t	c	0.9132	0.1223	0.0612	1.130093079	0.04577	8	++++-+-+
7	81739258	rs77959549	a	g	0.9132	0.1221	0.0612	1.129867083	0.04611	8	++++-+-+
7	81738903	rs2097914	t	c	0.0869	-0.1214	0.0612	0.885679617	0.04712	8	----+-+-
7	82008631	7:82008631:T_TG	i	r	0.4448	-0.0669	0.0338	0.935288726	0.04759	6	------++
7	81745621	7:81745621:TA_T	d	r	0.0859	-0.1203	0.0612	0.8866544	0.04927	8	----+-+-

Multi-ethnic meta-analysis IOP (Hysi et al., Nature Genetics, 2014 Oct;46(10):1126-30.												
CHR	BP	SNP	Allele 1	Allele 2	Freq1	Effect (beta)	StdErr	p-value	q_p-value	i2	n-studies	Direction
7	82011353	rs2299184	a	g	0.2024	0.0188	0.0312	0.546435	0.354773	0.08504	17	++++?--++++-+--+--
7	82011505	rs2299185	a	g	0.7913	-0.0108	0.0305	0.723717	0.268008	0.15845	17	----?++----+-++-++
7	82011839	rs6955149	a	g	0.7958	-0.0121	0.0310	0.695687	0.324138	0.11091	17	----?++----+-++-++
7	82013761	rs11971283	c	g	0.8698	-0.0224	0.0377	0.552393	0.520873	0	17	----?+----++-+--++
7	82013521	rs11982475	t	c	0.8744	-0.0213	0.0384	0.579035	0.367274	0.07444	17	--+-?+----++-++-++
7	82013724	rs10274659	t	c	0.7754	0.0026	0.0296	0.93056	0.225233	0.19556	17	-+--?++-+--+-++-++
7	82009164	rs10954678	c	g	0.4292	0.0091	0.0245	0.709253	0.295265	0.13260	18	++-++-++++++---+--
7	82009771	rs10808299	a	c	0.5741	-0.0147	0.0244	0.548053	0.265419	0.15734	18	-----+------+++-++
